# Herpes Zoster: A Rare Dermatosis in Childhood (About 25 Cases)

**DOI:** 10.1155/jotm/2286964

**Published:** 2025-03-04

**Authors:** Fatimazahra El Fatoiki, Yousra Habibi, Anas Saddik, Fouzia Hali, Soumya Chiheb

**Affiliations:** Department of Dermatology and Venereology, Hassan II Faculty of Medicine and Pharmacy, Ibn Rochd University Hospital Center, Casablanca, Morocco

**Keywords:** children, herpes zoster, viral dermatosis

## Abstract

**Introduction:** Herpes zoster is a viral dermatosis that occurs after reactivation of the varicella-zoster virus (VZV). The aim of this study is to illustrate the epidemiological and clinical aspects, as well as the complications, of herpes zoster in children.

**Materials and Methods:** This is a descriptive, prospective study of a series of 25 children followed for herpes zoster over a 3 year period in the dermatology department of CHU Ibn Rochd in Casablanca.

**Results:** There were 16 boys and 9 girls, with a mean age of 8.05 years. None of the patients had been vaccinated against varicella. Nine patients were immunocompromised. All patients were treated with antivirals, analgesics, antiseptics, and antibiotics (for seven infected patients). All patients had a favorable outcome with no sequelae.

**Discussion:** VZV belongs to the Herpesviridae family, an enveloped virus with a DNA genome. It has a particular affinity for the skin, nervous system, and lungs. Shingles is a rare disease in children, which typically follows a favorable course without sequelae. In children with shingles, if the history and physical examination are normal, laboratory testing for occult immunodeficiency or malignancy is not necessary. The diagnosis is primarily clinical. Except for ophthalmic forms, complicated cases, and immunocompromised patients, no specific treatment is required. In immunocompromised children, the infection is usually severe and disseminated, leading to high morbidity and mortality, and requires specific intravenous antiviral treatment.

**Conclusion:** Herpes zoster is a rare condition in children, typically evolving without sequelae.

## 1. Introduction

Herpes zoster (shingles) is a viral dermatosis that occurs after reactivation of the varicella-zoster virus (VZV), which remains latent in the dorsal sensory ganglia following primary varicella infection [1]. Herpes zoster in children is rare, but recent reports indicate an increase in the number of cases in pediatric populations [2]. The risk factors for herpes zoster in children are not well understood, but it is not associated with malignancies as it is in adults and can occur in otherwise healthy children without immunosuppression [3]. The particularity of this form of the disease in children is the predominance of general signs, the generally favorable course, and the rarity of postzosteralgia [4].

The aim of this study is to illustrate the epidemiological, clinical, and complication aspects of herpes zoster in children, based on a series of 25 pediatric cases.

## 2. Materials and Methods

This is a prospective and descriptive study of a series of 25 children followed in the dermatology department of CHU Ibn Rochd in Casablanca, Morocco, for herpes zoster over a 3 year period from March 23, 2020, to March 23, 2023. We included all patients under the age of 15 who presented with herpes zoster. Data were collected from patient medical records, including clinical characteristics and anthropo-demographic data (age: calculated from the year of birth to the consultation date, sex). We also collected data regarding comorbidities (immunosuppression: primary immunodeficiency, retroviral infection, neoplasia, hematological malignancies) and information related to the patients' evolution during a 1 year follow-up consultation.

### 2.1. Ethical Considerations

Informed consent from both the parents and children was obtained prior to inclusion in the study, while ensuring patient anonymity during data collection. Approval was obtained from the Ibn Rochd University Hospital Ethics Committee.

## 3. Results

**Figure 1:**
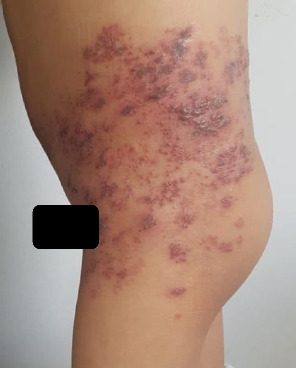
Intercostal shingles in an 11-year-old immunocompetent child.

**Figure 2:**
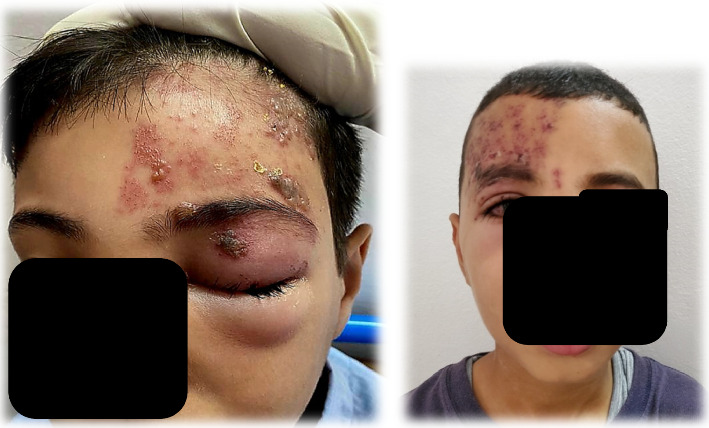
Ophthalmic shingles in a 7-year-old immunocompetent child.

There were 16 boys and 9 girls, with a mean age of 8.05 years (ranging from 3 to 15 years) (Table 1). The lesion locations were intercostal in 10 patients (Figure 1), on the upper or lower limbs in 4 patients, cervicofacial in 4 patients, ophthalmic in 4 patients (Figure 2), and genital in 2 patients. None of our patients had been vaccinated against varicella.

Nine patients were immunocompromised (3 with acute lymphoblastic leukemia, 2 with lymphoma, 2 with primary immunodeficiency, 2 with HIV) (Table 2). The lesions were typically erythematous vesicles in all cases, associated with necrotic and hemorrhagic lesions in 5 cases, and purulent vesicle content in 8 cases.

Varicella was reported in 20 of our patients. Only 5 patients did not have a clearly identified episode according to their parents. The primary infection may have gone unnoticed or could have been explained by a primary infection in the mother during the peripartum or perinatal period.

Our patients were treated with antivirals (oral aciclovir), analgesics, antiseptics, and antibiotics in 7 cases. For the nine immunocompromised patients, injectable antiviral (aciclovir) treatment was recommended. The treatment duration ranged from 8 to 15 days. All of our patients had a good outcome, with no sequelae. We ensured follow-up for a period of 1 year. No complications were observed, including no ocular complications in cases of herpes zoster ophthalmicus and no post-herpetic neuralgia.

## 4. Discussion

VZV belongs to the Herpesviridae family, an enveloped virus with a DNA genome. It has a particular affinity for the skin, nervous system, and lungs. After primary infection (chickenpox), the virus reaches the sensory ganglia via hematogenous and/or neurogenic routes from the skin or mucous membranes. Upon reactivation, it migrates along sensory nerve fibers to the skin [1].

Shingles is a rare condition in children, which usually has a favorable course with no sequelae. The incidence of shingles increases with age, although children who have had chickenpox during the first year of life (or in utero) have an increased risk of developing shingles. In the article Incidence of Herpes Zoster Among Children, Weinmann et al. report that the crude incidence rate for all subjects was 74 per 100,000 person-years, and the rate among vaccinated children was 38 per 100,000 person-years. The incidence of shingles is lower following varicella vaccination compared to natural infection. Shingles in children is often benign, post-herpetic neuralgia is rare, and antiviral treatment is generally not necessary. In a child with shingles, if the history and physical examination are normal, there is no need for laboratory testing to rule out occult immunodeficiency or malignancy [5].

Diagnosis is primarily clinical. Except for ophthalmic forms, complicated cases, and immunocompromised patients, no specific treatment is required. In immunocompromised children, the infection is usually severe and disseminated, leading to high morbidity and mortality, and requiring specific intravenous antiviral treatment [4, 6, 7].

The results of a Turkish study (Aktaş, Erdal, and Güvenç) conducted in 2019, which assessed the demographic and clinical characteristics of 60 cases of herpes zoster in children, align with the findings of our study, showing a male predominance (37 boys vs. 23 girls), and a mean age of 8 ± 4.93 years (Table 3). Of all cases, 46 had a history of varicella, and three patients had been vaccinated against varicella. Itching, observed in 48 subjects, was the most common symptom, while 38 subjects complained of pain. Acyclovir was prescribed as antiviral treatment in 33 cases. No complications developed in any of the cases [2].

In the article Complications of Herpes Zoster in Children, Kanamori et al. mention several complications, including facial paralysis, meningitis, uveitis, keratitis, acute retinal necrosis, pneumonia, and otitis interna. No complications were observed in our patients.

## 5. Conclusion

Herpes zoster is a rare disorder in children, generally with a favorable course and no sequelae. Diagnosis is primarily clinical. Herpes zoster can also affect immunocompetent children, but this infection should be investigated for underlying immunodeficiency, particularly neoplasia or primary or secondary immune deficiencies. The predominant lesion topography was intercostal, limb, and ophthalmic, with a clear male predominance. Treatment was based on antivirals, analgesics, and antiseptics. Complications are exceptional.

## Figures and Tables

**Table 1:** Demographic and clinical characteristics of patients with herpes zoster.

Demographic and clinical characteristics	Average age (years)	Gender	Vaccine	Complications
Aktas et al.	8 ± 4.93	0,61	3	0
Our study	8,05	0,64	0	0

**Table 2:** Antecedent of immunodeficiency.

Antecedent	Number of patients
HIV	2
Lymphoma	2
Leukemia	3
Primary immune deficiency	2

**Table 3:** Type of study and number of cases.

Study	Country	Study type	Number of cases
Aktas et al.	Turkey	Retrospective	60
Our study	Morocco	Prospective	25

## Data Availability

Data sharing not applicable to this article as no datasets were generated or analysed during the current study.
